# Rapid and Sensitive Lentivirus Vector-Based Conditional Gene Expression Assay to Monitor and Quantify Cell Fusion Activity

**DOI:** 10.1371/journal.pone.0010954

**Published:** 2010-06-03

**Authors:** Manuel A. F. V. Gonçalves, Josephine M. Janssen, Maarten Holkers, Antoine A. F. de Vries

**Affiliations:** Department of Molecular Cell Biology, Leiden University Medical Center, Leiden, The Netherlands; Institut Pasteur Korea, Republic of Korea

## Abstract

Cell-to-cell fusion is involved in multiple fundamental biological processes. Prominent examples include osteoclast and giant cell formation, fertilization and skeletal myogenesis which involve macrophage, sperm-egg and myoblast fusion, respectively. Indeed, the importance of cell fusion is underscored by the wide range of homeostatic as well as pathologic processes in which it plays a key role. Therefore, rapid and sensitive systems to trace and measure cell fusion events in various experimental systems are in demand. Here, we introduce a bipartite cell fusion monitoring system based on a genetic switch responsive to the site-specific recombinase FLP. To allow flexible deployment in both dividing as well as non-dividing cell populations, inducer and reporter modules were incorporated in lentivirus vector particles. Moreover, the recombinase-inducible transcription units were designed in such a way as to minimize basal activity and chromosomal position effects in the “off” and “on” states, respectively. The lentivirus vector-based conditional gene expression assay was validated in primary human mesenchymal stem cells and in a differentiation model based on muscle progenitor cells from a Duchenne muscular dystrophy patient using reporter genes compatible with live- and single-cell imaging and with whole population measurements. Using the skeletal muscle cell differentiation model, we showed that the new assay displays low background activity, a 2-log dynamic range, high sensitivity and is amenable to the investigation of cell fusion kinetics. The utility of the bipartite cell fusion monitoring system was underscored by a study on the impact of drug- and RNAi-mediated p38 MAPK inhibition on human myocyte differentiation. Finally, building on the capacity of lentivirus vectors to readily generate transgenic animals the present FLP-inducible system should be adaptable, alone or together with Cre/loxP-based assays, to cell lineage tracing and conditional gene manipulation studies *in vivo*.

## Introduction

Diverse biological phenomena involve fusion between either the same or different cell types (i.e. homotypic and heterotypic cell fusion, respectively). Notable examples include osteoclast and giant cell formation (macrophage fusion), fertilization (sperm-egg fusion), syncytiotrophoblast formation (cytotrophoblast fusion) and skeletal muscle development and regeneration (myoblast fusion). In addition, recent studies indicate that in parallel to these canonical cell fusion processes there are also those involving non-canonical or atypical cell fusion events. For example, it has been advanced that cancers may progress through heterotypic cell fusion between tumor cells and cells with tissue-infiltrating capacity such as macrophages [1], [2]. Another example is the occurrence of low-level heterotypic cell fusion between stem or progenitor cells from one tissue and parenchymal cells from another tissue especially under conditions disrupting the homeostasis of the recipient organ. The latter phenomenon stirred up the stem cell field by providing an alternative explanation to some of the early observations on stem cell plasticity whereby cells, in some cases, purportedly transdifferentiated, i.e. acquired a phenotype different from the cells of their tissue of origin [3]. In addition, cell-to-cell fusion is also a feature evolved by syncytium-forming viruses (e.g. human immunodeficiency virus [HIV] and measles) to facilitate spreading through their hosts [4], [5]. Of note, syncytium formation “as-a-means-to-spread” has been proposed as a process that could be exploited to increase the potency of oncolytic virus-based vectors [6]–[8]. Given its relevance in biology, flexible and sensitive assays to detect and measure cell-to-cell fusion are expected to have a broad applicability.

Cell fusion activity is often expressed in terms of the “fusion index”. The “fusion index” is obtained by estimating the frequency of nuclei associated with syncytia in relation to the total number of nuclei. Alternatively, putative fusion partners can be marked with different fluorescent proteins or dyes that produce a distinctive combinatorial spectral signature after fusion between the differently labeled cells takes place [9]. Also, species-specific antibodies as well as species- or gender-specific chromosome probes can be applied to detect fusion events between cells of different organisms or genders. These methods, however, are based on microscopic inspection and cell counting by different researchers and, as a consequence, they are fastidious, time-consuming and error-prone. This makes them suffer from a lack of high-throughput capabilities, accuracy and reproducibility. Additionally, “fusion index” estimations cannot discriminate *bona fide* cell fusion events from aborted cytokinesis.

To overcome these limitations more quantitative and reproducible cell fusion assays have been developed (for a review see [10]). Because the most direct method of demonstrating cell fusion is to ascertain mixing of cellular constituents of the interacting partners, these assays have in common the measurement of a new signal output only after such mixing occurs. The majority of quantitative cell fusion assays are based either on biochemical complementation or on transcription activation principles. The former rely on assembly of tetrameric complexes consisting of two *Escherichia coli* β-galactosidase subunits that *per se* are non-functional due to the deletion of crucial protein domains [11], whereas the latter depend on bacteriophage T7 RNA polymerase- or bacteriophage Cre recombinase-responsive reporter genes [10]. The Cre recombinase-based systems possess a characteristic arrangement of genetic elements (see below) and allow the detection of sporadic cell fusion events *in vivo* [12]. Of note, normally low Cre concentrations suffice to induce target DNA rearrangement with subsequent signal amplification being relayed via reporter gene transcriptional activity. In contrast, albeit potentially faster in reporting cell fusion events, the β-galactosidase complementation method require the component protein subunits to be present in high and, ideally, equimolar amounts to facilitate the assembly of catalytically active enzyme complexes.

This report describes the development and testing of a novel two-component transcription activation-dependent cell fusion assay based on the introduction into separate test cell populations of recombinase-encoding and recombinase-responsive transcription units. The recombinase-encoding expression unit directs the synthesis of FLPe, an enhanced version of the *Saccharomyces cerevisiae* site-specific recombinase FLP [13], whereas the recombinase-responsive transcription module contains a direct repeat of FLP recognition target (FRT) sites embedded within a unique arrangement of transcription elements that allows the formation of functional transgenes solely upon the generation of circular episomes (see below). This FLPe-responsive molecular switch, which differs from those usually applied in recombinase-activatable transgenes, was chosen to minimize reporter gene basal activity in the “off” state and to avoid chromosomal position effects, including transcriptional interference, on reporter gene expression in the “on” state. Importantly, to allow rapid and flexible deployment in dividing and non-dividing cells as well as in cells displaying a limited replicative life span (e.g. most primary cells), both components of the assay system were incorporated into lentivirus vector particles. This new conditional gene expression system was validated in an *ex vivo* differentiation model based on myoblasts from a Duchenne muscular dystrophy (DMD) patient [14] using reporter genes amenable to live- and single-cell imaging and to whole-population measurements. Finally, by deploying the bipartite cell fusion monitoring system together with short-hairpin RNAs (shRNAs) and the specific p38α/β inhibitor SB 203580 we showed the involvement of the p38 mitogen-activated protein kinase (MAPK) pathway in human myotube formation.

## Results and Discussion

### Experimental rationale and design

Chromosomal position effects imparted on transgenic DNA by flanking genomic sequences potentially complicate reliable quantification of recombinase-dependent transgene expression. This pitfall may be overcome by using clonal derivatives of the test cells or by targeted insertion of the recombinase-activatable transcription unit into specific chromosomal *loci*. However, both these approaches rely on the establishment and screening of single-cell clones which makes them laborious and time-consuming. Moreover, recombinant DNA transfer as well as selection and expansion of clones are not easily applicable to cells with a limited replicative life span like most primary mammalian cells. Furthermore, working with single-cell clones may yield biased results due to the possible existence of phenotypic heterogeneity in the test cell population.

**Figure 1:**
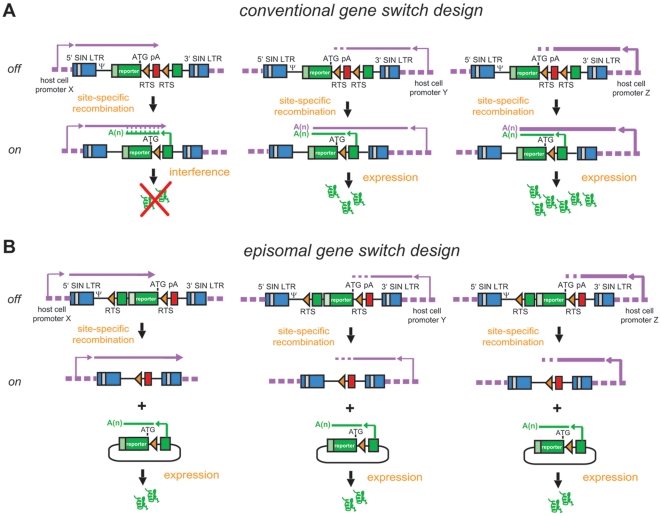
Structure and *modus operandi* of recombinase-responsive lentivirus vector-based gene switch modules with the conventional (A) or the episomal design (B). SIN LTR, SIN hybrid long terminal repeat composed of HIV-1 and RSV sequences; ψ, HIV-1 packaging signal; RTS, recombinase target site; pA, polyadenylation signal; dashed magenta lines, chromosomal DNA; magenta and green broken arrows, endogenous and exogenous promoter elements, respectively. See the main text section “Experimental rationale and design” for a detailed explanation of the figure.

Besides their prominent role in gene therapy research [15], lentivirus vectors have become widely used gene transfer tools due to their straightforward production and capacity to stably transduce both dividing and non-dividing cells with high efficiency. The versatility of lentivirus vector technology is further underscored by its successful deployment in establishing transgenic rodent lines [16], [17]. Lentivirus vectors achieve permanent genetic modification of transduced cells by integrating DNA copies of their genomes into the host cell's chromosomal DNA. Integration events, however, are not strictly random since the lentiviral pre-integration complexes preferentially target actively transcribed open reading frames (ORFs) [18], [19]. As a result, individual DNA integrants become subjected to different chromatin environments and associated regulatory *cis*-acting elements leading to positional effects on transgene expression (**Fig. 1A**). In the context of a recombinase-dependent conditional gene expression system with a conventional organization of genetic elements these chromatin positional effects may include *in cis* (depicted) and *in trans* transcriptional interference on reporter gene expression (**Fig. 1A**). To minimize these effects we designed a lentivirus vector encoding a recombinase-dependent gene switch module with an arrangement of its genetic elements as illustrated in **Fig. 1B**. Of note, this lentivirus vector-based conditional gene expression system contains the unidirectional murine *metallothionein* gene polyadenylation signal (pA) previously shown to block cryptic transcription of a promoterless ORF located downstream of it [20]. This element, as well as the reporter ORF and the rabbit *β-globin* pA associated with it, are oriented in the opposite direction to that of the lentivirus vector 5′ long terminal repeat (LTR)-driven transcription to prevent interference with the generation of full-length vector genomes during particle production. FLPe-mediated site-specific DNA recombination and ensuing reporter gene activation is concomitant with a transition of the expression unit from a chromosomal to an episomal context. This topological change should eliminate possible negative or positive effects of chromosomal *cis*-acting regulatory elements on reporter gene expression (**Fig. 1B**). Putative DNA integrant-specific reporter activity in the “on” state due to epigenetic modifications acquired by the transgenic sequences following their host cell genome insertion is expected to be avoided neither by the traditional nor by the new gene switch design.

### Generation and testing of lentivirus vectors bearing recombinase-responsive reporter genes whose activation is dependent on episome formation

**Figure 2:**
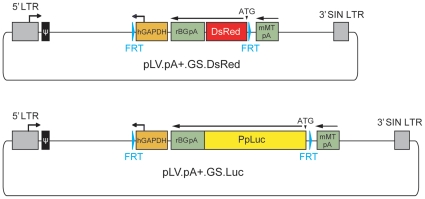
Diagram of the lentivirus vector shuttle plasmids pLV.pA+.GS.DsRed and pLV.pA+.GS.Luc. Grey box with broken arrow, 5′ hybrid long terminal repeat (LTR) containing Rous sarcoma virus and HIV-1 sequences; Grey box without broken arrow, 3′ SIN LTR; ψ, HIV-1 packaging signal; Cyan arrowheads, FLP recognition target (FRT) sites; orange box with broken arrow, human *glyceraldehyde-3-phosphate dehydrogenase* (hGAPDH) gene promoter; Large and small green boxes, rabbit *β-globin* and murine *metallothionine* gene pAs (rBGpA and mMTpA, respectively); red box, *DsRed.T4-N1* ORF from *Discosoma sp*. (DsRed); yellow box, *luciferase* ORF from *Photinus pyralis* (PpLuc). All genetic elements are drawn to scale.

We started by testing whether lentivirus vector particles encoding a FLP-dependent gene switch with the structural organization depicted in **Fig. 1B** could be generated and, following indicator cell transduction, express the reporter gene in a recombinase-dependent manner. To this end, LV.GS.DsRed particles produced by using the lentivirus vector shuttle plasmid pLV.pA+.GS.DsRed (**Fig. 2**) were added onto myoblasts derived from a satellite cell of a DMD patient [14] at 0, 3, 9 and 15 transducing units (TU)/cell. The pLV.pA+.GS.DsRed construct encodes an enhanced version of the red fluorescent protein (RFP) of *Discosoma sp.* designated DsRed.T4-N1 [21].

**Figure 3:**
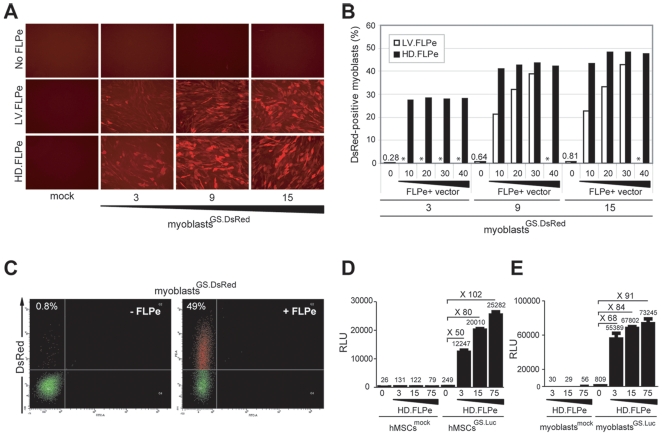
Functional testing of the lentivirus vectors LV.GS.DsRed and LV.GS.Luc. (**A**) Direct fluorescence microscopy of human myoblasts not exposed (mock) or exposed to 3 different doses of LV.GS.DsRed (i.e. 3, 9 and 15 TU/cell) in the absence (no FLPe) or in the presence of FLPe (LV.FLPe or HD.FLPe). (**B**) Flow cytometric analysis of human myoblasts transduced with 3 different dosages of LV.GS.DsRed (i.e. 3, 9 and 15 TU/cell) and subsequently exposed to increasing amounts of FLPe-encoding viral vectors (i.e. LV.FLPe [open bars] and HD.FLPe [solid bars]) or not. Experimental conditions not tested are marked by an asterisk (*). (**C**) Dot plot representation of *DsRed.T4-N1* expression in human myoblasts stably transduced with LV.DsRed (myoblasts^GS.DsRed^) in the absence (-FLPe) or presence (+FLPe) of HD.FLPe. (**D and E**) Luminometric analysis of luciferase activity in lysates derived from hMSCs or myoblasts mock-treated (**D and E**; hMSCs^mock^ and myoblasts^mock^, respectively) and from hMSCs or myoblasts LV.GS.Luc-transduced (**D and E**; hMSCs^GS.Luc^ and myoblasts^GS.Luc^, respectively) that were or were not exposed to different amounts of HD.FLPe particles. Graph bars shows mean ± standard error of the mean (*n = *3). RLU, relative light units.

Next, these cells were either mock-infected or incubated with a single dose of the FLPe-encoding lentivirus vector LV.FLPe or of the FLPe-encoding high-capacity adenovirus vector HD.FLPe.F50 [22] (hereinafter referred to as HD.FLPe). Three days post-infection direct fluorescence microscopy revealed that synthesis of RFP was dependent on the presence of FLPe and that more red fluorescent myoblasts were observed in cultures initially exposed to higher doses of LV.GS.DsRed (**Fig. 3A**). In another experiment, parallel cultures of DMD myoblasts transduced with LV.GS.DsRed at 0, 3, 9 and 15 TU/cell were exposed to increasing amounts of HD.FLPe (i.e. 0, 10, 20, 30 and 40 gene switch-activating units [GSAU]/cell) or LV.FLPe (i.e. 0, 10, 20, 30 TU/cell). Flow cytometric analyses performed at 3 days after infection confirmed FLPe-mediated transgene activation and an LV.GS.DsRed dose-dependent increase in the frequency of RFP-positive cells (**Fig. 3B**). Dot plots corresponding to the flow cytometric analyses of DMD myoblasts exposed to or not exposed to HD.FLPe, are depicted in **Fig. 3C** (right and left panel, respectively). Importantly, in the absence of FLPe, the frequency of RFP-positive cells in myoblast cultures transduced with increasing amounts of LV.GS.DsRed remained below 1% (range: 0.28–0.81%).

On the basis of the results described above, we constructed pLV.pA+.GS.Luc to allow quantification of cell fusion activity in whole cell populations. This lentivirus vector shuttle plasmid contains the *luciferase* gene from *Photinus pyralis* (**Fig. 2**) whose product permits the detection of gene expression within a very wide linear range by using simple bioluminescence assays. These assays measure the visible light emitted during the enzymatic conversion of luciferin to oxyluciferin. Lentivirus vector particles were generated by using the shuttle plasmid pLV.pA+.GS.Luc. The functionality of the LV.GS.Luc particles generated with the aid of pLV.pA+.GS.Luc was evaluated by transducing primary human mesenchymal stem cells (hMSCs) [9] and human myoblasts [14] at 30 TU/cell. Mock-transduced hMSCs and mock-transduced human myoblasts served as negative controls. Subsequently, cultures of these two different indicator cell types were exposed to the FLPe-encoding adenovirus vector HD.FLPe at 3, 15 and 75 GSAU/cell [22]. Data shown in **Figs. 3D and 3E** clearly demonstrate that robust *luciferase* gene expression in both hMSCs (**Fig. 3D**) and human myoblasts (**Fig. 3E**) is dependent on the presence of FLPe. For each of the HD.FLPe doses applied, the fold increase in luciferase activity in comparison to cells transduced exclusively with LV.GS.Luc, was similar for both cell types (i.e. 50, 80 and 102 for hMSCs versus 68, 84 and 91 for myoblasts at HD.FLPe doses of 3, 15 and 75 GSAU/cell, respectively).

Taken together these data indicate that (i) the specific arrangement of genetic elements in the FLP-responsive reporter gene-containing shuttle plasmids is compatible with the synthesis and packaging of lentivirus vector genomic RNA and (ii) host cell promoter-driven reporter gene expression is contained as evident from the low basal expression levels measured following proviral vector DNA integration into the genome of the indicator cells.

### Quantification of cell fusion in *ex vivo* cultures of human muscle progenitor cells

To investigate the suitability of the present lentivirus vector-based inducible reporter gene system to detect and quantify cell fusion events we exploited an *ex vivo* cellular differentiation model based on human myoblasts. In this system, activation of the terminal myogenic differentiation program is simply accomplished by withdrawing mitogens from the culture medium. This triggers the homotypic fusion of the mononucleated muscle progenitor cells resulting in a time-dependent accumulation of nuclei in syncytial structures called myotubes.

**Figure 4:**
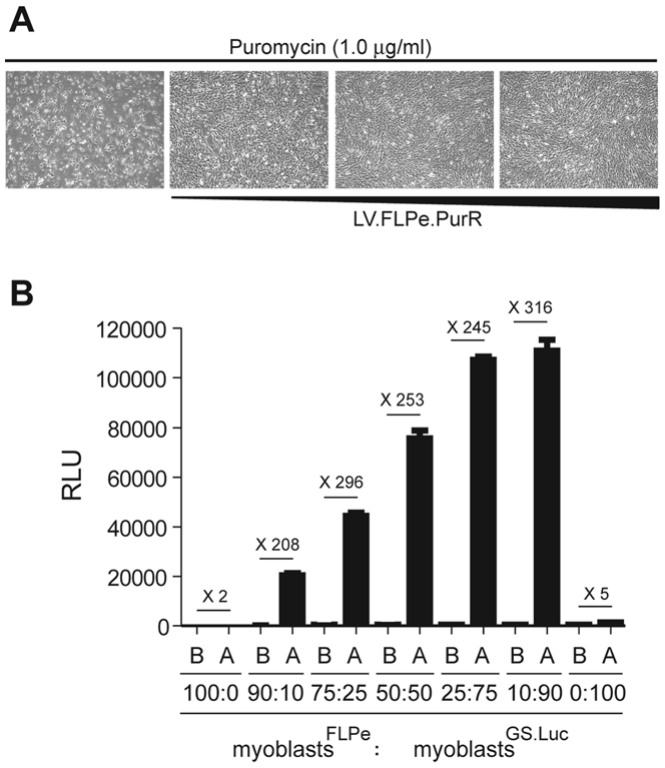
Fusion-dependent reporter gene activation in an *ex vivo* human skeletal muscle cell differentiation system. (**A**) Establishment of FLPe-positive human myoblast cultures. Phase-contrast microcopy of human myoblasts initially incubated with 0, 30, 300 and 900 µl of cleared culture supernatant from LV.FLPe.PurR-producing 293T cells. Micrographs were acquired 7 days posttransduction and 5 days after the addition of puromycin to a final concentration of 1.0 µg/ml. (**B**) Luminometric analysis of cell lysates derived from co-cultures of human myoblasts stably transduced with LV.FLPe.PurR or with LV.GS.Luc (myoblasts^FLPe^ and myoblasts^GS.Luc^, respectively). The two types of human myoblast populations were mixed at the indicated ratios and luciferase activity was measured before (B) and after (A) myogenic differentiation. Bars represent mean ± standard error of the mean (*n = *3). RLU, relative light units.

FLPe-encoding human myoblasts were generated through transduction with the lentivirus vector LV.FLPe.PurR followed by selection of genetically modified cells using puromycin. Five days after the initiation of puromycin-mediated selection, an LV.FLPe.PurR dose-dependent increase in cell survival could be discerned (**Fig. 4A**). Repeated passaging of LV.FLPe.PurR-transduced human myoblasts in the presence of puromycin revealed no apparent differences in the growth rate between cell cultures exposed to the different amounts of FLPe-encoding vector tested (not shown). Thus, we selected myoblasts transduced with the highest LV.FLPe.PurR dose (hereinafter referred to as myoblasts^FLPe^) to carry out the subsequent experiments. In parallel, reporter gene-modified test cells (hereinafter referred to as myoblasts^GS.Luc^) were generated by transducing human myoblasts with LV.GS.Luc at a multiplicity of infection of 30 TU/cell.

To determine whether the lentivirus vector-based inducible reporter gene system displays (i) low basal activity in the presence of FLPe-positive cells and (ii) high fusion-dependent induction factors, we mixed myoblasts^FLPe^ with myoblasts^GS.Luc^ at different ratios (i.e. 90∶10, 75∶25, 50∶50, 25∶75 and 10∶90) (**Fig. 4B**). Cultures consisting exclusively of myoblasts^FLPe^ (i.e. 100:0) or of myoblasts^GS.Luc^ (i.e. 0∶100) served as negative controls (**Fig. 4B**). The extent of myogenic differentiation observed in each of the myoblast cultures after mitogen withdrawal was similar (not shown). Bioluminescence measurements immediately prior to as well as after the completion of the myoblast-myotube differentiation schedule clearly revealed a differentiation/fusion-dependent increase in luciferase activity. Indeed, reporter gene expression levels more than 2 orders of magnitude above those in non-differentiated cells were consistently measured exclusively in co-cultures of myoblasts^FLPe^ and myoblasts^GS.Luc^ (**Fig. 4B**).

### Analysis of cell fusion kinetics in *ex vivo* cultures of human muscle progenitor cells

**Figure 5:**
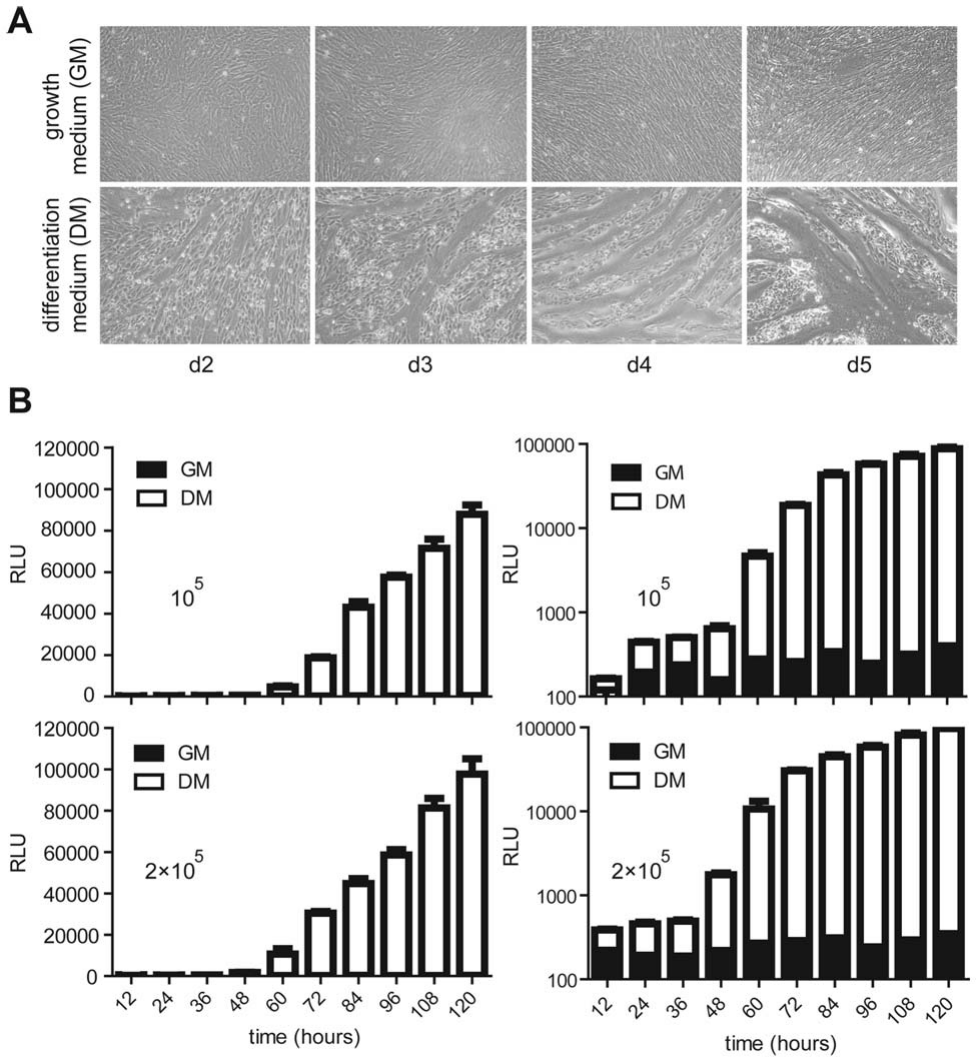
Time-dependent reporter gene activation in an *ex vivo* human skeletal muscle cell differentiation system. (**A**) Phase contrast microscopy of co-cultures containing myoblasts^FLPe^ and myoblasts^GS.Luc^ at a 1∶1 ratio after incubation for 2, 3, 4 or 5 days in growth medium (upper panels) or in differentiation medium (lower panels). (**B**) Luminometric analysis of cell lysates prepared at 12-hour intervals from co-cultures initiated with 5×10^4^ myoblasts^FLPe^ and with 5×10^4^ myoblasts^GS.Luc^ (upper panels) or with 10^5^ myoblasts^FLPe^ and with 10^5^ myoblasts^GS.Luc^ (lower panels). The cells were either maintained in growth medium (solid bars) or in differentiation medium (open bars). The relative light units (RLU) are plotted on linear (left panels) and logarithmic (right panels) scales. Bars correspond to mean ± standard error of the mean (*n = *3).

As previously mentioned, upon induction of myogenic differentiation a gradual accumulation of myotubes of growing size and with an increasing number of nuclei can be observed in cultures of myoblasts as a consequence of ongoing cell fusion. Conversely, parallel cultures kept under normal growth medium are mostly devoid of myotubes. We asked whether this myogenic differentiation phenomenon could be followed as a function of time by deploying the lentivirus vector-based conditional gene expression system presented above (**Fig. 5**). To this end, myoblasts^FLPe^ were mixed with myoblasts^GS.Luc^ at a 1∶1 ratio using two different total amounts of cells (i.e. 10^5^ and 2×10^5^). The resulting co-cultures were subsequently either kept in growth medium or were exposed to differentiation medium for different periods of time (**Fig. 5A**, upper and lower panels, respectively). Luminometric analyses of lysates from cells maintained in differentiation medium revealed a time-dependent increase in luciferase activity, which correlated with the time-dependent rise in the frequency and size of myotubes. In addition, consistent with the previous findings (**Fig. 4B**), parallel co-cultures of myoblasts^FLPe^ and myoblasts^GS.Luc^ maintained in regular growth medium did not show a time-dependent rise in *luciferase* expression despite the increase in cell numbers due to mitosis (**Fig. 5B**, solid bars in right panels). These data suggest that the newly developed lentivirus vector-based conditional gene expression system can be deployed not only to quantify muscle cell differentiation activity but should also be useful to investigate factors that positively or negatively affect the kinetics of this process.

### Determining the sensitivity of the lentivirus vector-based inducible reporter gene assay to detect cell fusion in *ex vivo* cultures of differentiating human myoblasts

Earlier data showed that transgene expression in co-cultures of myoblasts^GS.Luc^ and myoblasts^FLPe^ increased with increasing fractions of myoblasts^GS.Luc^ (**Fig. 4B**) suggesting that the number of gene switch modules available for FLP-mediated recombination was limiting. This is consistent with the idea that relatively few molecules of recombinases such as Cre or FLP suffice to bring about site-specific recombination and that, necessarily, the more *luciferase* copies are present in the bipartite gene expression system the higher the likelihood for reporter protein synthesis to occur.

**Figure 6:**
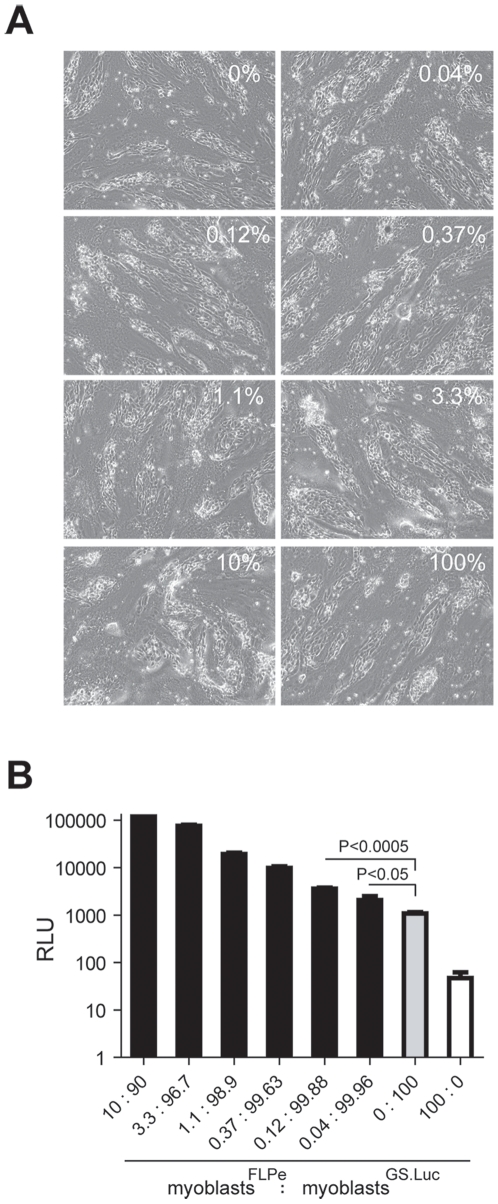
Probing the sensitivity of the lentivirus vector-based conditional gene expression assay to detect myoblast fusion. (**A**) Phase contrast microscopic images of cultures started with a total number of 2×10^5^ myoblasts. The pictures were taken after a 5-day incubation in differentiation medium. These cultures consisted exclusively of myoblasts^GS.Luc^ (bar labeled 0∶100) or of myoblasts^FLPe^ (bar labeled 100:0) or, contained mostly myoblasts^GS.Luc^ (90–99.96%) spiked with different amounts of myoblasts^FLPe^ (i.e. 0.04, 0.12, 0.37, 1.1, 3.3 and 10%). (**B**) Luciferase activity in cultures initiated with a total number of 2×10^5^ myoblasts and exposed for 5 days to differentiation medium. The cultures were composed exclusively of myoblasts^GS.Luc^ (grey bar) or of myoblasts^FLPe^ (white bar) or contained myoblasts^GS.Luc^ (90–99.96%) together with low proportions of myoblasts^FLPe^ (i.e. 0.04, 0.12, 0.37, 1.1, 3.3 and 10%). Cumulative data are expressed as means ± standard error of the mean (*n = *3). *P* values resulting from the comparison of relevant experimental groups were determined using an unpaired one-tailed Student's t-test. *P* values <0.05 were considered significant. RLU, relative light units.

Thus, to determine the minimum percentage of myoblasts^FLPe^ that still leads to a measurable fusion-dependent signal output, we set up an experiment in which the frequency of myoblasts^FLPe^ was titrated on a constant total amount of 2×10^5^ myoblasts. The fraction of myoblasts^FLPe^ present in co-cultures of myoblasts^FLPe^ and myoblasts^GS.Luc^ was varied in a series by a factor of 3. Of note, no obvious differences in the frequency and size of myotubes were observed between the co-cultures containing different ratios of myoblasts^FLPe^ and myoblasts^GS.Luc^ (**Fig. 6A**). Chemiluminescence measurements performed after the regular differentiation period showed that a frequency of myoblasts^FLPe^ as low as 0.04% could still give rise to a luciferase signal above that measured in cultures containing exclusively myoblasts^GS.Luc^ (i.e. background [grey bar]) (**Fig. 6B**). These results bode well for the deployment of the current lentivirus vector-based conditional gene expression system in instances in which fusion events are rare. Even so, ways by which to optimize the current set up can be envisioned. These include (i) flanking of the gene switch module by insulator sequences to block host cell enhancer activity on reporter gene expression during the proviral vector stage, (ii) placing transgene expression under the control of a promoter stronger than that of the human *glyceraldehyde-3-phosphate dehydrogenase* gene and (iii) replacing the current RFP- and *Photinus pyralis* luciferase-coding sequences by those of reporter proteins with higher specific activity like DsRed-Express2 [23] and *Gaussia princeps* luciferase [24]. Of note, the latter reporter protein in its secreted [24] or membrane-anchored [25] form should permit adjustment to novel experimental read-outs with the added advantage of displaying a higher sensitivity than other commonly used luciferases. Finally, analysis of cell fusion kinetics may profit from the deployment of destabilized versions of luciferase or other reporter proteins.

### Pharmacological and genetic interference with the p38 MAPK pathway and its impact on the human myoblast fusion process

Various studies, mostly in murine and rat cells, have shown that the p38 MAPK pathway plays a pivotal role in skeletal myogenesis through, amongst others, phosphorylation-dependent activation of myogenic transcription factors and its ability to induce chromatin remodeling of skeletal muscle-specific gene promoters. A recent study deploying neonatal myoblasts derived from mice in which the *p38α*, *p38β*, *p38γ* and *p38δ* genes were individually knocked out pinpointed p38α as the most crucial p38 MAPK in this muscle cell differentiation system [26]. In fact, murine muscle progenitor cells lacking p38β or p38δ did not present any apparent defect in their fusion ability whereas those without p38γ displayed a delayed fusion phenotype [26].

**Figure 7:**
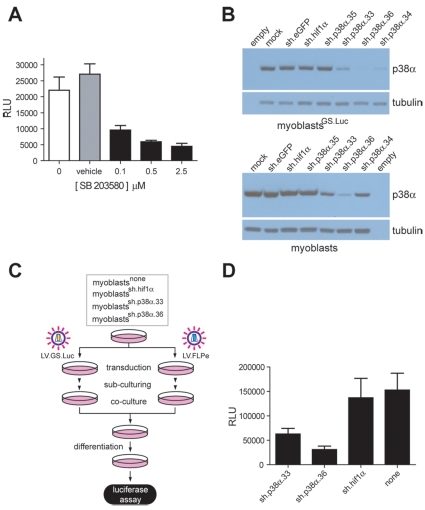
Pharmacological and genetic inhibition of the p38 MAPK pathway and its impact on human myotube formation *in vitro*. (**A**) Luminometric analysis of cell lysates generated from co-cultures initiated with 10^5^ myoblast^FLPe^ and 10^5^ myoblast^GS.Luc^ cells and exposed for 3 days to differentiation medium (white bar), to differentiation medium supplemented with 0.1, 0.5 and 2.5 µM SB 203580 (black bars) or to differentiation medium containing a final concentration of vehicle equivalent to that applied to co-cultures incubated with 2.5 µM SB 203580 (grey bar). Cumulative data are presented as means ± standard deviations (*n = *3). RLU, relative light units. (**B**) Western blot analysis of p38α levels in protein lysates of parental myoblasts and myoblasts^GS.Luc^ (mock) and of myoblasts and myoblasts^GS.Luc^ stably transduced with shRNA modules designed to down-regulate expression of *eGFP* (sh.eGFP), *hif1α* (sh.hif1α) and human *p38α* (sh.p38α.35, sh.p38α.33, sh.p38α.36 and sh.p38α.34). The α- and β-tubulins served as loading control. (**C**) Diagram outlining the experimental set-up applied to investigate the impact of post-transcriptional down-regulation of *p38α* expression on human myocyte fusion (see text for details). (**D**) Quantification through chemiluminescence of myoblast fusion activity in co-cultures consisting of a 1∶1 mixture of FLPe- and GS.Luc-encoding myoblasts either not transduced (none) or stably transduced with shRNAs sh.p38α.33, sh.p38α.36 or sh.hif1α. Data were derived from a minimum of 3 and a maximum of 6 different experiments and are presented as means ± standard error of the mean. RLU, relative light units.

To determine whether the bipartite conditional gene expression system could be used to establish the involvement of the p38 MAPK pathway during the differentiation of myocytes from human adults, we deployed the p38α/β-specific inhibitor SB 203580 or shRNAs targeting *p38α* transcripts. Co-cultures consisting of myoblasts^FLPe^ and myoblasts^GS.Luc^ kept in regular differentiation medium or in differentiation medium supplemented with either SB 203580 (0.1, 0.5 and 2.5 µM) or vehicle only, revealed a clear drug-dependent reduction in myoblast fusion activity as determined by chemiluminescence measurements (**Fig. 7A**). Subsequently, by using lentivirus vector-mediated gene transfer and puromycin selection, we screened on the background of myoblasts and myoblasts^GS.Luc^ cells, polyclonal lines encoding shRNAs designed for post-transcriptional down-regulation of *p38α* (4 shRNAs), *eGFP* (1 shRNA) or murine *hypoxia inducible factor 1α* (*hif1α*) (1 shRNA) expression. Screening of the relative amounts of p38α protein synthesized in parental myoblasts and myoblasts^GS.Luc^ as well as in the various shRNA-encoding polyclonal cell populations derived from them was carried out by Western blot analysis. Results depicted in **Fig. 7B** show that myoblast and myoblast^GS.Luc^ lines encoding shRNAs sh.p38α.33 and sh.p38α.36 displayed consistent *p38α* knock-down. To measure the impact of *p38α* knock-down on human myocyte fusion, the experimental set-up outlined in **Fig. 7C** was followed. Cultures of parental myoblasts, myoblasts^sh.hif1α^, myoblasts^sh.p38α.33^ and myoblasts^sh.p38α.36^ were divided and transduced either with LV.FLPe or with LV.GS.Luc. After sub-culturing, the resulting FLPe-encoding cell populations were mixed at a 1∶1 ratio with their respective GS.Luc-containing counterparts (i.e. myoblasts^sh.p38α.36+FLPe^ were mixed with myoblasts^sh.p38α.36+GS.Luc^ and so forth). Luminometry-based cell-to-cell fusion measurements following the exposure of the various co-cultures to differentiation medium for 4 days showed that specific down-regulation of *p38α* expression in human muscle progenitor cells results in impaired cell fusion (**Fig. 7D**). These results together with those presented in **Fig. 7A** indicate that the p38 MAPK pathway is involved in terminal differentiation of human myoblasts *in vitro*.

In summary, we introduce herein a novel cell fusion assay based on the activation of a reporter gene by recombinase-dependent episome formation. We showed that, following lentivirus vector-mediated gene transfer, the reporter units display low basal activity and a strict dependency on the FLP recombinase for their activation. These features combined with the ability of lentivirus vectors to swiftly generate transgenic mammals [16], [17] might render the present FLP-responsive system useful to investigate cell fusion *in vivo* and, alone or together with Cre/loxP-based assays, assist in cell-lineage and conditional gene expression studies. Finally, in this post-genomic era, the challenge is to go from primary sequence data to phenotypic associations that are underpinned by functional read-outs. Key to this ongoing endeavor is the continuous development of robust high-throughput cell-based assays to link genotypes to specific phenotypes. Related to this, we expect the current gene switch system combined with high-throughput screening of chemical, small interfering/short hairpin RNA and cDNA libraries [27]–[30] to serve as a powerful methodology to identify molecules and pathways involved in the ill-understood process of mammalian muscle cell fusion. As an initial proof-of-principle we used the conditional gene expression assay presented herein to demonstrate by two different approaches the involvement of the p38 MAPK pathway during terminal differentiation of human myocytes *in vitro*.

## Materials and Methods

### Recombinant DNA

DNA constructions were carried out by using established procedures [31] or following the instructions provided with specific reagents. In brief, construct pLV.CMV.hMyoD.eGFP [32] (GenBank accession number: EU048697) was digested with Bsu15I and Bsp1407I (both from Fermentas). The 6.3-kb DNA segment containing the plasmid backbone was extracted from gel and its 3′ recessed ends were filled in with Klenow polymerase (Fermentas). In parallel, pGS.pA+.DsRed [20] was digested with SmaI, HindIII and SspI. The resulting 2.4-kb fragment was extracted from gel and treated with Klenow polymerase. The 6.3-kb and 2.4-kb DNA fragments were ligated to each other giving rise to construct pLV.pA+.GS.DsRed. To construct pLV.pA+.GS.Luc, pLV.pA+.DsRed and pGL3.Basic (Promega) were digested with BcuI plus MluI and HindIII plus XbaI, respectively. Next, the 8.0-kb pLV.pA+.GS.DsRed fragment without the *DsRed.T4-N1* ORF and the luciferase-coding DNA segment from pGL3.Basic were extracted from gel, blunt-ended with Klenow polymerase and ligated to each other, yielding pLV.pA+.GS.Luc.

All ligation mixtures were introduced into the GeneHogs strain of *Escherichia coli* (Invitrogen) and large-scale plasmid purifications were performed using JETSTAR 2.0 Plasmid Maxiprep kits (Genomed) according to the manufacturer's instructions. The nucleotide sequences of pLV.pA+.GS.DsRed and pLV.pA+.GS.Luc can be retrieved by using GenBank accession numbers GU253312 and GU253313, respectively. In addition, the annotated sequences corresponding to the FLPe-encoding lentivirus vector shuttle plasmids pLV.FLPe and pLV.FLPe.PurR can be accessed via GenBank accession codes GU253315 and GU253314, respectively.

### Cells

The culture conditions for the hMSCs [9] as well as the culture and differentiation conditions for the human myoblasts [14] have been described previously [9], [14], [33].

### Viral vectors

The production and titration of the FLPe-encoding high-capacity adenovirus vector HD.FLPe have been detailed elsewhere [22]. The vesicular stomatitis virus G protein-pseudotyped self-inactivating (SIN) HIV type 1 (HIV-1)-based vectors LV.FLPe, LV.FLPe.PurR, LV.GS.DsRed and LV.GS.Luc were generated in 293T cells with the aid of the packaging plasmids psPAX2 (Addgene) and pLP/VSVG (Invitrogen) as specified before [32]. To concentrate and purify lentivirus vector particles, producer cell supernatants were layered onto 5-ml cushions of 20% (wt/vol) sucrose (Merck) in phosphate-buffered saline (PBS) and centrifuged at 15,000 rotations per minute (rpm) for 2 hours at 10°C in an SW28 rotor (Beckman Coulter). Prior to ultracentifugation, producer cell supernatants were subjected to filtration through 0.45-µm pore-sized cellulose acetate filters (Pall). The VSV-G-pseudotyped HIV-1-based vectors LV.sh.eGFP, LV.sh.hif1α, LV.sh.p38α.33, LV.sh.p38α.34, LV.sh.p38α.35 and LV.sh.p38α.36 were generated in 293T cells by using pLKO.1-puro clones SHC005, TRCN0000054448, TCRN0000000509, TCRN0000000510, TCRN0000000511 and TCRN0000000513 (Sigma-Aldrich; MISSION TCR1 library), respectively, together with the aforementioned packaging constructs. LV.sh.eGFP and LV.sh.hif1α encode shRNAs directed against, respectively, *eGFP* and *hif1α* (4 nucleotide mismatches with that of the human homologue) transcripts. Cells transduced with LV.sh.eGFP or LV.sh.hif1α served, together with mock-infected myoblasts and myoblasts^GS.Luc^, as negative controls in *p38α* expression knock-down experiments.

Physical particle titers were determined using the RETRO-TEK HIV-1 p24 Antigen ELISA kit (ZeptoMetrix) following the instructions provided by the manufacturer. Titers of lentivirus vector stocks expressed in terms of TU/ml were derived by using a conversion factor of 10 TU per pg of p24 protein.

### Cell transductions

Human myoblasts and hMSCs were seeded at a density of 2×10^5^ and 3×10^4^ cells per well of 24-well plates (Greiner Bio-One), respectively, and were exposed overnight (*circa* 20 hours) to viral vectors in a humidified-air 10% CO_2_ atmosphere. Next, the cell monolayers were rinsed multiple times with large volumes of PBS after which fresh medium was added. Mock infections were carried out in parallel cultures by using exclusively the respective cell type's culture medium.

### Co-culture establishment and maintenance

Co-cultures containing 10^5^ or 2×10^5^ cells were established in wells of 24-well plates by mixing myoblasts^FLPe^ with myoblasts^GS.Luc^ in the specified ratios After an incubation period of 48 to 72 hours the growth medium was either substituted by differentiation medium or by fresh growth medium. Unless otherwise indicated, the differentiation was allowed to proceed for 5 days after which the cultures were processed for analysis.

### Small-molecule drug inhibition of the p38 MAPK pathway in human myoblast cultures

Myoblasts^FLPe^ and myoblasts^GS.Luc^ were seeded in a 1∶1 ratio at a final total density of 2×10^5^ cells per well of 24-well plates. After 3 days of co-culture, the growth medium was replaced with differentiation medium, differentiation medium containing 0.1, 0.5 or 2.5 µM of 4-[4-(4-fluorophenyl)-2-(4-methylsulfinylphenyl)-1*H*-imidazol-5-yl]pyridine (SB 203580; Promega) or differentiation medium containing the same final concentration of SB 203580's vehicle dimethyl sulphoxide (Hybri-Max; Sigma-Aldrich) as the co-cultures incubated with 2.5 µM of the p38α/β inhibitor. The different types of media were replenished every 24 hours. Generation of cell lysates and luciferase measurements 3 days after the addition of the various types of differentiation media were carried out as described under “Luciferase assay”.

### Western blot analysis of shRNA-mediated knock-down of *p38α* gene expression

The various shRNA-encoding polyclonal lines were established by exposing myoblasts and myoblasts^GS.Luc^ for 24 hours to clarified supernatants of LV.sh.eGFP, LV.sh.hif1α, LV.sh.p38α.33, LV.sh.p38α.34, LV.sh.p38α.35 and LV.sh.p38α.36 producer cells. Subsequently, these polyclonal lines were sub-cultured in the presence of 0.5 µg/ml of puromycin. Control cells corresponding to parental myoblasts and myoblasts^GS.Luc^ did not survive this drug selection regimen.

Monolayers of myoblasts and myoblasts^GS.Luc^ either mock-transduced or stably transduced with the shRNA-encoding sequences targeting *eGFP*, *hif1α* and *p38α* transcripts (2×10^5^ cells) were rinsed with ice-cold PBS and dissolved in 100 µl of ice-cold lysis buffer composed of 20 mM Tris-HCl (pH 7.6), 150 mM NaCl, 0.1% sodium dodecyl sulfate (SDS), 0.5% sodium deoxycholate, 1% Nonidet P-40 and 10% glycerol plus a cocktail of protease inhibitors (Complete Mini, Roche Applied Science). Next, the lysates were spun for 8 min at 4°C at 20,800× *g*. Prior to sample loading, the total protein concentration in the various cell lysates was determined by using the BCA Protein Assay Kit (Thermo Fisher Scientific) and a bovine serum albumin standard curve. Instructions corresponding to the microplate procedure, as provided by the manufacturer, were followed except for the use of 3 instead of 25 µl of cell lysate. Also, the absorption measurements were performed at 545 instead of 562 nm in a Wallace 1420 VICTOR 3 multilabel plate reader (PerkinElmer). Next, equal protein amounts were subjected to electrophoresis through an SDS-12% polyacrylamide gel after which they were transferred onto an Immobilon-P membrane (Millipore) by electroblotting overnight. After incubation with blocking solution (10 mM Tris-HCl [pH 8.0], 150 mM NaCl and 0.05% Tween-20 [TBST]) supplemented with 10% (w/v) non-fat dry milk powder (Elk, Campina), the membranes were incubated overnight at 4°C with the rabbit anti-human p38α affinity-purified IgG antibody AF8691 (R&D systems) diluted 1∶3000 in blocking solution. Subsequently, the membranes were washed 4 times with TBST at room temperature and subjected to a 3-hour incubation with horseradish peroxidase-conjugated goat-anti-rabbit IgG secondary antibody (SC-2009; Santa Cruz Biotechnology) diluted 1∶5000 in blocking solution. Detection of α/β-tubulin proteins by using a rabbit anti-human α/β-tubulin affinity-purified IgG antibody (2148; Cell Signaling Technology) at a dilution of 1∶5000, provided for loading controls. Finally, the membranes were rinsed and processed for protein detection by chemiluminescence as described elsewhere [22].

### Effect of shRNA-mediated knock-down of *p38α* gene expression on human muscle cell fusion

Myoblasts, myoblasts^sh.hif1α^, myoblasts^sh.p38α.33^ and myoblasts^sh.p38α.36^ were seeded at a density of 1.5×10^5^ cells in wells of 24-well plates. After an overnight incubation period, the cells were exposed for *circa* 16 hours to either LV.FLPe.PurR (15 TU/cell) or LV.GS.Luc (30 TU/cell). After being expanded via sub-culturing, the resulting FLPe-encoding cells were mixed at a 1∶1 ratio with their respective GS.Luc-containing counterparts (i.e. 10^5^ myoblasts^sh.p38α.36+FLPe^ were mixed with 10^5^ myoblasts^sh.p38α.36+GS.Luc^ and so forth). Following 3 days in growth medium, the four different types of co-cultures were incubated in differentiation medium for 4 additional days, after which, reporter gene expression was determined as specified below.

### Luciferase assay

Cell monolayers in wells of 24-well plates were washed with 1 ml of PBS and were lysed by incubation for 20 minutes in 200 µl of ice-cold lysis buffer consisting of 25 mM Tris-phosphate (pH 7.8), 2 mM dithiothreitol, 2 mM EDTA (pH 8.0), 10% glycerol and 1% Triton X-100. During the incubation period with the lysis buffer, the plates were swirled approximately every 5 minutes to homogenize the constituents present in the wells. Subsequently, the recovered cell lysates were spun at 14,000 rpm for 3 minutes at 4°C in a microcentrifuge (Eppendorf 5417R). The resulting supernatants were stored at −20°C until analysis. The luciferase activity present in one-tenth of each supernatant was measured with the aid of a Wallace 1420 VICTOR 3 multilabel plate reader loaded with luciferase assay substrate (Promega) reconstituted in luciferase assay buffer (Promega) as specified by the supplier.

### Fluorescence microscopy

The equipment and methods used for the light microscopy analyses have been described before [22], [32]–[34].

### Flow cytometry

The flow cytometry instrument and procedures have been specified elsewhere [22], [34].
